# Draft Genome Sequence of *Lactobacillus kosoi* NBRC 113063, Isolated from Kôso, a Japanese Sugar-Vegetable Fermented Beverage

**DOI:** 10.1128/MRA.01173-18

**Published:** 2018-11-21

**Authors:** Tai-Ying Chiou, Wataru Suda, Kenshiro Oshima, Masahira Hattori, Chiaki Matsuzaki, Kenji Yamamoto, Tomoya Takahashi

**Affiliations:** a Department of Biotechnology and Environmental Chemistry, Kitami Institute of Technology, Kitami, Hokkaido, Japan; b Laboratory for Microbiome Sciences, Center for Integrative Medical Sciences, RIKEN, Yokohama, Kanagawa, Japan; c Department of Computational Biology, Graduate School of Frontier Sciences, The University of Tokyo, Kashiwa, Chiba, Japan; d Research Institute for Bioresources and Biotechnology, Ishikawa Prefectural University, Nonoichi, Ishikawa, Japan; e ARSOA Research & Development Center, AOB Keioh Group Corporation, Hokuto, Yamanashi, Japan; Louisiana State University

## Abstract

Lactobacillus kosoi NBRC 113063 is a fructophilic species isolated from kôso, a Japanese sugar-vegetable fermented beverage. The draft genome sequence of Lactobacillus kosoi NBRC 113063 is useful for understanding the carbohydrate metabolism of fructophilic lactic acid bacteria.

## ANNOUNCEMENT

Kôso is a popular Japanese sugar-vegetable beverage that is made from a wide-ranging mixture of vegetables (or fruits) and sugars. The fermentation of kôso occurs spontaneously based on a complex microbial community contained in the raw materials (1, 2). In a previous study, we used next-generation sequencing to examine the time-dependent changes in the bacterial community in kôso. We found that an unclassified Lactobacillus, which appeared to be a difficult-to-culture species, predominates (2). By using a dilution-to-extinction technique, the species was successfully isolated and proposed as Lactobacillus kosoi sp. nov. (3).

For the sequencing effort, de Man, Rogosa, and Sharpe (MRS) broth (Difco Laboratories) supplemented with 10% (wt/vol) d-fructose was used as the medium for cultivation of L. kosoi NBRC 113063 (= BCRC 81100). After 2 days of cultivation at 30°C under an aerobic condition (unmodified atmosphere), the preparation of genomic DNA was performed following the procedure in our previous report (2).

An Illumina MiSeq system was used to perform whole-genome sequencing of L. kosoi. A total of 1,960,663 reads were assembled into 67 contigs using Newbler v2.8 (Roche). The contig *N*_50_, average contig size, and largest contig size were 38,792 bp, 21,266 bp, and 112,389 bp, respectively. The resulting draft genome sequence is 1,424,862 bp with an average read coverage of 382.0× and an average G+C content of 30.52%. Annotation was performed with Prokka 1.11 (http://www.vicbioinformatics.com/software.prokka.shtml) (4) with default settings, and the results show that the draft genome of L. kosoi NBRC 113063 contains 1,376 candidate open reading frames, 1 repeat region, 1 transfer-messenger RNA (tmRNA) gene, 3 rRNA genes, and 56 tRNA genes.

In our previous report (3), L. kosoi did not grow in MRS broth but grew well in the MRS broth which was supplemented with 5% d-fructose. L. kosoi was thus regarded as a fructophilic lactic acid bacterium (5). However, the mechanism of being fructophilic is still not clear. In the draft genome of L. kosoi NBRC 113063, we found 3 putative genes for fructokinase, which phosphorylates fructose as part of the fructose metabolic process. Since L. kosoi possesses 3 fructokinase genes, it appears that fructose is preferred in the carbon assimilation. One glucokinase gene and 1 galactokinase gene were also found in the genome, which suggests that L. kosoi might be able to utilize glucose and galactose under certain conditions. The phosphotransferase (PTS) system regulates carbohydrate metabolism in bacteria (6); however, the necessary genes were not found in L. kosoi. This may be one of the reasons that the carbohydrate metabolism in L. kosoi NBRC 113063 was not successfully characterized using the API 50 CHL kit (3). In addition, the genes related to the CRISPR-Cas system were found in L. kosoi. Since the CRISPR-Cas system is important in bacterial immune systems and can be applied for genome engineering (7), the genomic information obtained will be helpful for further understanding L. kosoi.

### Data availability.

The genome sequence of L. kosoi NBRC 113063 has been deposited in DDBJ/EMBL/GenBank under accession number BEXE00000000. The version described in this paper is version BEXE01000000. The data discussed here can be accessed at https://www.ncbi.nlm.nih.gov/genome/?term=Lactobacillus+kosoi. The sequence reads of L. kosoi NBRC 113063 have been deposited in DDBJ under accession number DRR152974.

## References

[1] Chiou T-Y, Oshima K, Suda W, Hattori M, Takahashi T 2016 Draft genome sequence of *Lactobacillus farciminis* NBRC 111452, isolated from kôso, a Japanese sugar-vegetable fermented beverage. Genome Announc 4:e01514-15. doi:10.1128/genomeA.01514-15. 26769925 PMC4714107

[2] Chiou T-Y, Suda W, Oshima K, Hattori M, Takahashi T 2017 Changes in the bacterial community in the fermentation process of kôso, a Japanese sugar-vegetable fermented beverage. Biosci Biotechnol Biochem 81:403–410. doi:10.1080/09168451.2016.1249449. 27800754

[3] Chiou T-Y, Suda W, Oshima K, Hattori M, Matsuzaki C, Yamamoto K, Takahashi T 2018 *Lactobacillus kosoi* sp. nov., a fructophilic species isolated from kôso, a Japanese sugar-vegetable fermented beverage. Antonie Van Leeuwenhoek 111:1149–1156. doi:10.1007/s10482-018-1019-7. 29353462

[4] Seemann T 2014 Prokka: *rapid prokaryotic genome* annotation. Bioinformatics 30:2068–2069. doi:10.1093/bioinformatics/btu153. 24642063

[5] Endo A, Futagawa-Endo Y, Dicks LMT 2009 Isolation and characterization of fructophilic lactic acid bacteria from fructose-rich niches. Syst Appl Microbiol 32:593–600. doi:10.1016/j.syapm.2009.08.002. 19733991

[6] Siebold C, Flükiger K, Beutler R, Erni B 2001 Carbohydrate transporters of the bacterial phosphoenolpyruvate: sugar phosphotransferase system (PTS). FEBS Lett 504:104–111. doi:10.1016/S0014-5793(01)02705-3. 11532441

[7] Horvath P, Barrangou R 2010 CRISPR/Cas, the immune system of bacteria and archaea. Science 327:167–170. doi:10.1126/science.1179555. 20056882

